# How do they know: “instinct,” seasonality, hormones, or what?

**DOI:** 10.3325/cmj.2022.63.101

**Published:** 2022-02

**Authors:** Charles H. Calisher

“*Celestial reckoning. That’s how [Canada geese] go … by the stars. That’s how they find the ponds of Texas. Scientists study their ways, dissecting and inducing. The answers will elude them. It’s magic, and no one can argue me otherwise, at age ten or four decades later*.” (Robert James Waller; author, photographer, and musician)

Well, it is not magic. We simply do not completely understand everything in nature. That alone is a good thing. It would be terrible if we knew everything. Nothing to learn (schools at all levels would be for technical training, not investigating), nothing to do, no one to teach, no new ideas, nothing to talk about at cocktail parties.

I unabashedly, even proudly, admit that I, like many people, know a great deal. I have lived a long time, attended some good schools, worked at places that encouraged, even required, continuous learning, paid attention to details, made initial discoveries and worked hard. However, much of what I know is trivial. I can speak for hours at cocktail parties, fascinating those listeners who might have nothing else to do or too much to drink. I also can speak for hours with colleagues and students, write book chapters (which are outdated as soon as they appear in print), and even present formal lectures (with clever and entertaining PowerPoint slides) at all levels of educational institutions, fascinating students who had not heard of diseases, viruses, vaccines, mosquito and tick parts, and associated minutiae. Still, proud of all these accumulated factoids, I have no idea of the origin of the occurrences, the cause of the actions, and the reasons that some things act as they do. I am not ashamed of this ignorance, I just would like to know more but, unfortunately, I do not have the time in my busy life to learn everything.

For example:

1. HOW do plants know when to sprout and to flower? Almost every year, crop plants, garden plants, even most house plants send out buds, small lateral or terminal protuberances on the stems of vascular plants that may develop into a flower, leaf, or shoot. Buds arise from meristem tissue. The meristem is a type of plant tissue consisting of undifferentiated cells capable of cell division, much like teenagers. Cells in the meristem can develop into all the other tissues and organs that occur in plants. These cells continue to divide until a time when they differentiate and then lose the ability to divide. In temperate climates, trees form resting buds that are resistant to frost in preparation for winter. Wheat, usually *Triticum aestivum*, does the same thing and, remarkably, is somewhat protected from freezing by bacteria. Flower buds are modified leaves.

Stunted silage corn is called pineapple corn, because the tight leaves make it look more like a pineapple plant. Soybean plants flip their leaves over to reduce photosynthesis and thus the need for water, giving them a paler green appearance. “It should be six, seven, eight foot tall,” said the farmer, looking down at the stunted plants at his feet, their normally floppy leaves rolled tight against their stalks to conserve water in the summer heat.

Knowing all this is not very helpful in answering my question! I know that plants bud but what I want to know is HOW do the plants “know” when the time is right for them to bud? In fact, they do not know, otherwise they might not cause misery for farmers who lose their crops when a spring freeze kills the buds. Plants do not have “common sense” as we know it, they respond to water, light and dark, cold and heat, the presence of chemicals, genes, and other factors. Plants use an internal time-keeping mechanism known as the circadian clock to measure changes in day length. The circadian clock regulates the timing of the specific photoreceptor for flowering. That is the way plants sense differences in day length.

Nevertheless, something definitive is known about plant blooming. Flowers know when to bloom because of a gene named “Apetala 1,” which triggers the reproductive development of the plant, telling it when it is time to begin blossoming. That’s it; a single master gene is all that is required to make a plant begin producing flowers. So, it is in their genes but that is not sufficiently specific, so it also is not helpful in answering my question.

At our home, we have two different plantain lilies (*Hosta* spp) that the previous owners of our house kindly left to our care. We do nothing to maintain them. One is a small plant that produces simple, unattractive blooms each year. The other is a much larger plant that has produced beautiful, tall, lily-like flowers each year until this one. This year it has produced its usual considerable leaf system but no blooms. These two plants are no more than 10 cm apart in the garden but one has obviously not responded to its usual stimulants and preferred to put its energy into leaves, not blooms. If this is anything like the responses of mammals who can somehow foretell weather conditions far in advance (https://www.almanac.com/content/can-animals-predict-weather-animal-proverbs), it may not be a good sign. They are not paying attention to their hormones, which is always a good idea, at least for plants.

Fascinatingly, Amos Dolbear devised a mathematical formula to calculate the relationship between the air temperature (in degrees Celsius) and the rate at which crickets (*Oecanthus niveus*) chirp. Dolbear’s law (https://en.wikipedia.org/wiki/Dolbear%27s_law)

Tc = 10 + N 60 – 40

____________

7

where Tc = temperature in degrees Celsius and N = number of chirps per minute. Useful, if you are participating in a trivia contest but HOW do they do this? Why they do this is another question and even less useful in understanding HOW.

2. Much the same could be said for mammals. HOW do they know when to breed? Some breed in the fall (the definition of which differs from place to place) and the female reproductive systems are such (short or long gestation periods) that they give birth in the spring (early or late, short or long) when the weather is gentle enough to allow the offspring to survive it.

I suspect that human mammals look at their checking accounts or count the number of potatoes stored in their root cellars and decide when it is time to enlarge their families. (Sometimes they do this as the opposite of what Nobel Laureate Richard Feynman said: “Science is like sex: sometimes something useful comes out, but that is not the reason we are doing it.”)

3. Birds in the northern hemisphere, having the ability to fly to the Canary Islands, Mexico, or other warm places (who can blame them?), spend their winters, or otherwise non-optimum seasons, breeding and then either staying where they are in the pleasant conditions surrounding them or fly off to another nice place to raise their chicks. Birds in the southern hemisphere do the same thing, but at opposite times of year. Initiation of migration is related to the length of daylight and daily temperature and humidity ranges, the positions of the sun and stars, and whatever else they sense as making the proper conditions for migration but HOW do they do that? What does “sense” mean? Some birds have magnetite-based receptors above their nostrils which help them to use Earth's magnetic field as a compass. HOW does their geomagnetic genius tell them when to fly? Do birds ask themselves, “Is it time to leave?“ Of course not.

4. Our dogs, and probably all other domesticated canids, know when it is time to feed them, even when daylight savings time is initiated annually, take only one day to readjust their internal clocks to daylight savings time, know when to go to bed at night (even if we do not), and know when to jump into my lap and block my view of the TV when I am watching a particularly key part of an athletic event. HOW do they do this so accurately?

Members of all migrating animals use a variety of cues to determine when it is time to migrate. Some migrations are triggered by external cues, such as changing daylight hours or temperature. Other animals rely on internal cues like fat reserves or instinct, while others respond to multiple cues occurring at the same time. Migrating at a specific time is labeled “obligate migration.” Using cues from environmental conditions, such as food supplies and warming temperatures, is known as “facultative migration.” None of the papers I have read answer HOW they do this, what the molecular mechanism is. Thus, the words “instinct,” “hormonal responses,” “choice” (a biologically ridiculous term), “cues,” “obligate migration,” “facultative migration,” and other non-explanatory words and terms are not ultimately helpful in explaining what is expressed as “instinct.”

Certainly, these are not all coincidences. Do I need to go back to school to answer all these HOWs? I have asked people who are unquestionable experts in various fields, including genetics and wildlife biology, but they do not seem to know. Perhaps I am asking the wrong people or perhaps the HOWs are not known. Perhaps the answer to “HOW?” is the totality of a wide variety of small characteristics making the outcome obvious. Albert Einstein said, “Not everything that can be counted counts, and not everything that counts can be counted.” Who am I to argue with Einstein?

Still, I wish I could turn this essentially philosophical question into one that could be a more accurate and specific one. But, as comedian Woody Allen said, “I'm astounded by people who want to “know” the universe when it's hard enough to find your way around Chinatown.”

